# Buffalo milk transcriptome: A comparative analysis of early, mid and late lactation

**DOI:** 10.1038/s41598-019-42513-2

**Published:** 2019-04-12

**Authors:** Reena Arora, Anju Sharma, Upasna Sharma, Yashila Girdhar, Mandeep Kaur, Prerna Kapoor, Sonika Ahlawat, Ramesh Kumar Vijh

**Affiliations:** ICAR-National Bureau of Animal Genetic Resources, Karnal, 132001 Haryana India

## Abstract

The expression of genes and their regulation during lactation in buffaloes remains less understood. To understand the interplay of various genes and pathways, the milk transcriptome from three lactation stages of Murrah buffalo was analyzed by RNA sequencing. The filtered reads were mapped to the Bubalus bubalis as well as Bos taurus reference assemblies. The average mapping rate to water buffalo and Btau 4.6 reference sequence, was 75.5% and 75.7% respectively. Highly expressed genes (RPKM > 3000), throughout lactation included CSN2, CSN1S1, CSN3, LALBA, SPP1 and TPT1. A total of 12833 transcripts were common across all the stages, while 271, 205 and 418 were unique to early, mid and late lactation respectively. Majority of the genes throughout lactation were linked to biological functions like protein metabolism, transport and immune response. A discernible shift from metabolism in early stage to metabolism and immune response in mid stage, and an increase in immune response functions in late lactation was observed. The results provide information of candidate genes and pathways involved in the different stages of lactation in buffalo. The study also identified 14 differentially expressed and highly connected genes across the three lactation stages, which can be used as candidates for future research.

## Introduction

Lactation is a complex process which involves several physiological changes in the body, from development of mammary tissue to synthesis and secretion of milk. The anatomy of mammary glands as well as physiology of lactation differs across species^1^. Depending on the number of days from parturition, the lactation process is divided into early (14–100 days), mid (100–200 days) and late (more than 200 days) in buffalo. The milk yield and composition are influenced not only by nutrition, environmental factors, breed, age and season, but also by the stage of lactation^2–4^. Physiological and biological knowledge of lactation has led to improved management aspects resulting in increased milk production^5^. The synthesis and secretion of milk by the mammary gland involves a large number of genes. Comprehensive information of the molecular events, together with physiology of lactation will enhance our understanding of the process. The expression of genes and their regulation during lactation in buffaloes remains less understood, although buffaloes are the largest contributors to milk production in India, with a population of 109 million^6^. Comparison of the different stages of lactation may lead to identification of novel genes or transcripts that regulate lactation.

RNA sequencing offers an efficient and comprehensive portrayal of the expression of genes in a given tissue. Several studies have reported the discovery of differentially expressed as well as new genes in lactating mammals^5,7–10^. Although the transcriptomic data of several tissues of swamp buffalo is available^11^, little progress has been made on milk transcriptomics of water buffalo. The miRNA profile of buffalo milk is available^12^ and the cattle SNP Chip has been used for gene mining in dairy buffaloes of Brazil^13^. A comprehensive milk transcriptome profile will provide an insight into the dynamics of gene expression during lactation in buffaloes. Therefore, the aim of the present study was to identify different genes and pathways involved in the early, mid and late lactation stages of Murrah buffalo. The milk somatic cells were used in this study for transcriptome analysis, as they represent the mammary gland tissue and provide a non invasive source of RNA^14^. The study will improve our understanding of the genes and their interactions involved in the lactation process in buffaloes.

## Results

Four animals were selected in the early, mid and late lactation stage. All the animals were managed and fed as per the national code of practices for management of dairy animals in India^15^. These animals were multiparous with milk production ranging from 7–8 litres per day. The stage of lactation, age and milk yield of the animals is provided in Table S1.

### Summary of RNA seq data

**Figure 1:**
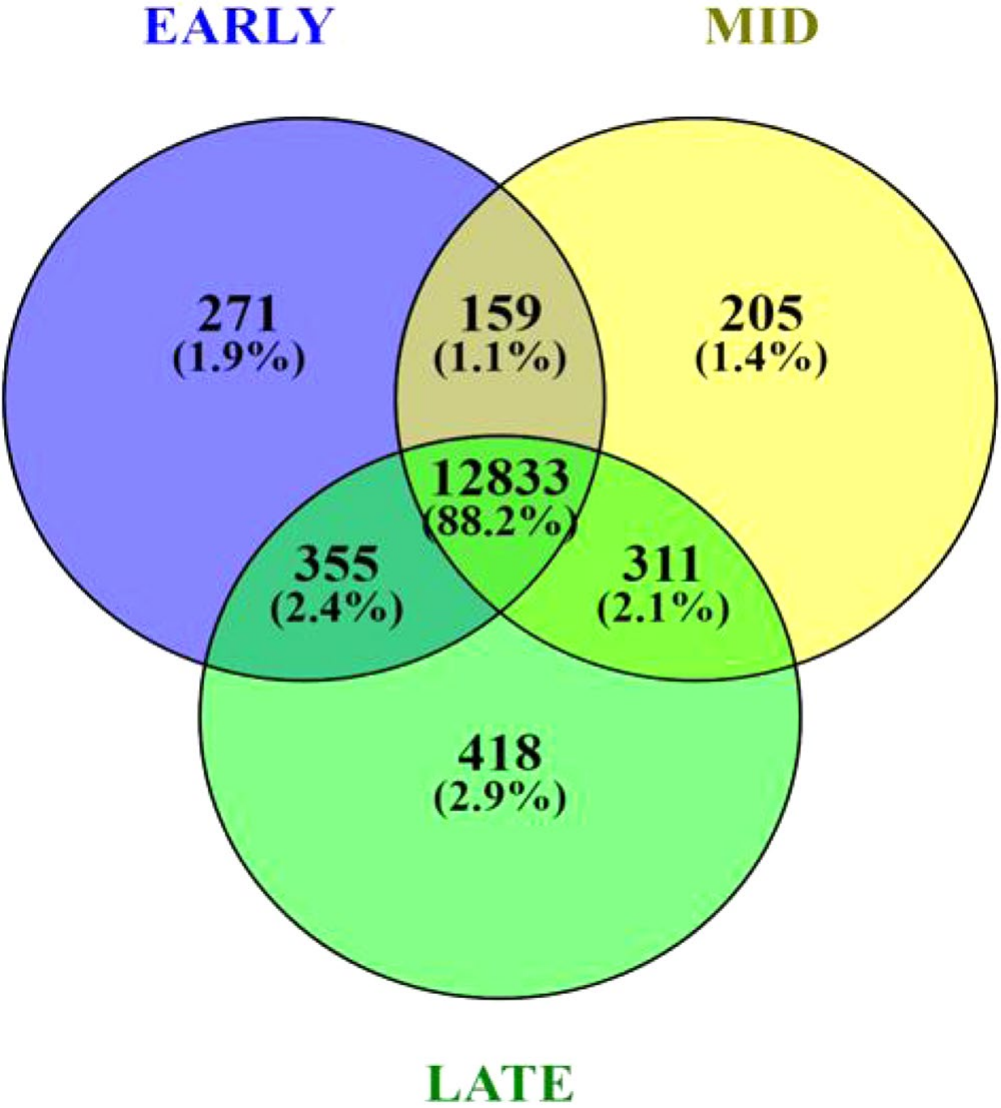
Venn diagram depicting distribution of transcripts across early, mid and late lactation stages in Murrah buffalo.

The average number of reads for each library of early (4), mid (4) and late (4) lactation was 70732975, 90585670 and 85196433, respectively. This Transcriptome Shotgun Assembly project has been deposited in GenBank with accession GGRC00000000.1, under BioProject PRJNA453843. The average mapping rate to water buffalo^16^ and Btau 4.6 reference sequence, across all the stages was 75.5% and 75.7% respectively. The uniquely mapped reads across early, mid and late stage were higher against *Bubalus bubalis* reference sequence than *Bos taurus* sequence (Table 1). Based on a threshold of >0.01 RPKM, 13618, 13508 and 13917 genes were observed in our dataset for early, mid and late lactation respectively. A total of 12833 transcripts were common across all the stages, while 271, 205 and 418 were unique to early, mid and late lactation respectively (Fig. 1). The number of genes with RPKM ≥ 500 was 120 for early, 119 for mid and 113 for late lactation.

**Table 1:** Summary of Read mapping statistics.

Sample	Total Reads	Total Mapped read	Unmapped Reads	Unique Reads	Mapping % (with *Bubalus bubalis*)	Mapping % (with *Bos Taurus*, Btau 4.6)
Buffalo1	98,060,582	80,273,720	17,786,862	5,126,351	81.86	77.77
Buffalo2	73,845,150	55,047,804	18,797,346	3,023,949	74.54	75.15
Buffalo3	63,252,478	55,103,964	8,148,514	3,284,575	87.12	85.09
Buffalo4	47,773,688	34,995,911	12,777,777	2,011,440	73.25	70.99
Buffalo5	88,926,132	71,705,483	17,220,649	4,372,871	80.63	79.06
Buffalo6	86,709,746	65,571,012	21,138,734	3,780,594	75.62	76.29
Buffalo7	80,978,704	62,982,821	17,995,883	3,972,074	77.78	76.94
Buffalo8	105,728,098	73,073,466	32,654,632	3,964,845	69.11	75.01
Buffalo9	108,754,608	75,890,698	32,863,910	4,762,890	69.78	74.23
Buffalo10	48,109,684	33,163,383	14,946,301	2,012,562	68.93	69.96
Buffalo11	114,278,262	84,782,139	29,496,123	5,190,874	74.19	73.84
Buffalo12	69,643,178	50,537,211	19,105,967	2,906,952	72.57	74.14

### Gene expression profile

**Figure 2:**
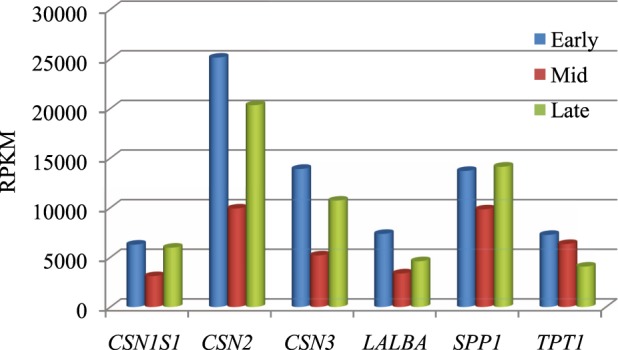
Higher expression genes, with RPKM > 3000, across three stages of lactation in Murrah buffalo.

**Figure 3:**
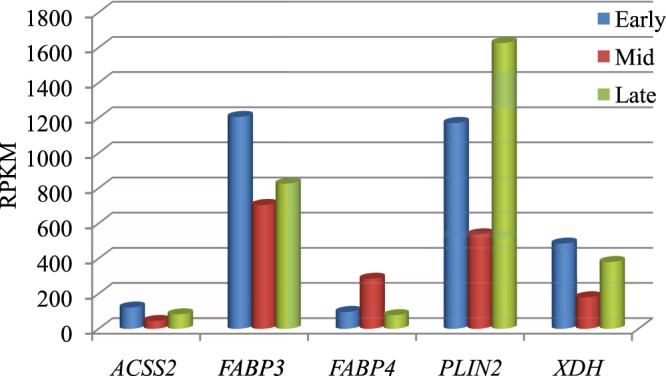
Expression of genes associated with milk fat, across the three lactation stages in buffalo.

Highly expressed genes (RPKM > 3000) throughout lactation included *CSN2*, *CSN1S1*, *CSN3*, *LALBA*, *SPP1* and *TPT1* (Table S2). Except *TPT1*, all genes had highest expression in the early stage, decreased in the mid stage and increased again in the late stage of lactation. The expression of *TPT1* declined from early to late stage. *SPP1* had higher RPKM value in the late stage, as compared to early and mid stage (Fig. 2). Besides these genes, *PAEP*, *B2M* and *FTHI* showed high expression levels (RPKM ≥ 5000), when Btau 4.6 was used as reference assembly. Genes associated with milk fat, having RPKM > 50 in any one stage, were *ACSS2*, *FABP3*, *FABP4*, *PLIN2*, and *XDH* (Fig. 3).

### Functional enrichment of genes across different stages of lactation

**Figure 4:**
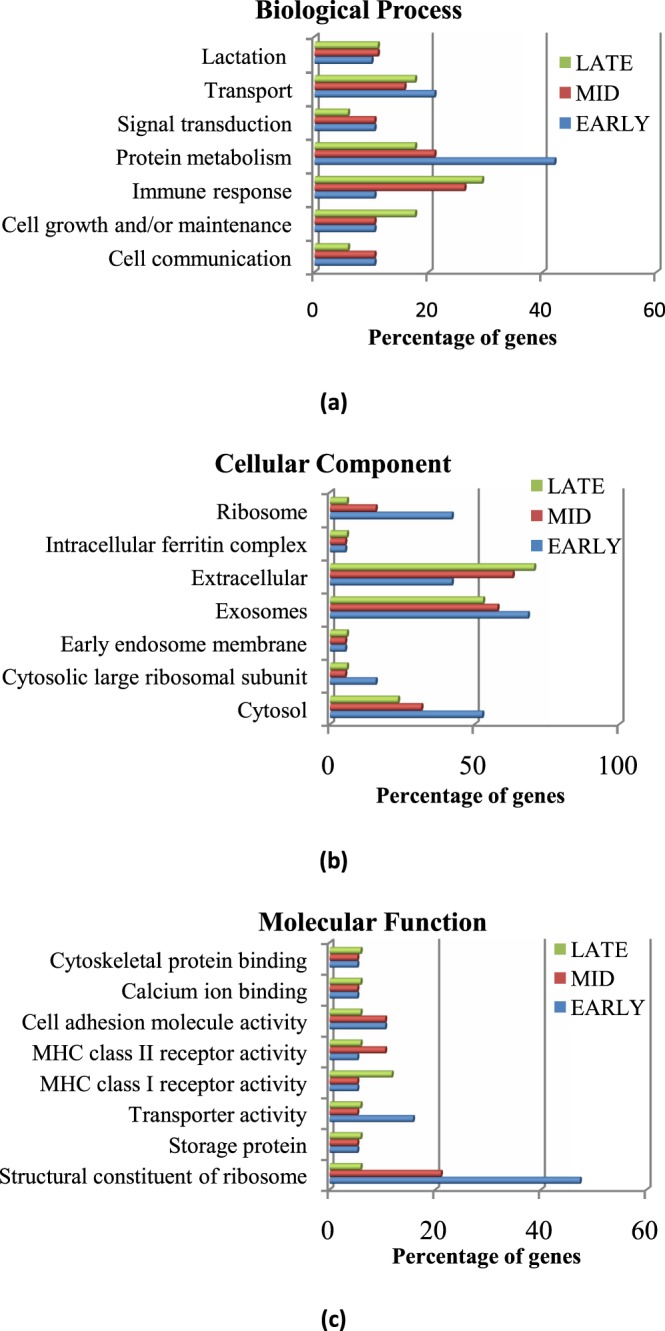
Gene ontology terms for (**a**) biological process (**b**) cellular components (**c**) molecular function for top 20 expressed genes in early, mid and late lactation stages of buffalo.

The highly expressed, top 20 genes (RPKM > 1800) from each stage of lactation were classified according to the gene ontology (GO) terminology. Although enrichment of several GO terms was similar across all stages, some marked differences were observed between early and late stages. Protein metabolism was the major biological process in the early stage, while immune response increased with progression of the lactation. Cell growth and maintenance, transport, signal transduction, lactation and cell adhesion were equally enriched in early and mid stages. The proportion of genes involved in cell growth, maintenance and transport increased during the late stage, whereas cell communication declined (Fig. 4a). Ribosome and cytosol were the main GO terms enriched for cellular components in the early stage whereas exosome and extracellular region were enriched in late stage (Fig. 4b). The defining term for molecular function in the early stage of lactation was structural constituent of ribosome, while storage protein, transporter activity, calcium ion binding, MHC Class I and II receptor activity were equally enriched across all the stages (Fig. 4c).

### Pathway analysis

The top 20 genes, with highest expression were analyzed for major biological pathways involved in each stage of lactation (Table S3). The number of significant pathways identified were 35, 91 and 79 for early, mid and late lactation respectively (p < 0.05). The early stage had 83% terms that were common with the mid stage, while 58% of the pathways of mid stage were also observed in the late stage. Diabetes, antigen presentation, Nef mediated downregulation/modulation of MHC class I complex, cell surface expression and endosomal/vacuolar pathways were common across all the three stages. Some of the significant pathways enriched in both early and mid lactation stages were ribosome, gene expression, GTP hydrolysis, insulin pathway and developmental biology. Pathways enriched in the mid and late lactation were Class I PI3K signaling events, Glypican pathway, insulin pathway etc. Although the pathways across the different stages were common, the percentage of genes involved in each pathway varied from stage to stage.

### Differentially expressed (DE) genes across early, mid and late lactation

Pairwise comparison was done between the 3 stages of lactation for identification of differentially expressed genes. Genes with log_2_ fold change (FC) ≥ 2.0 and p_adj_ ≤ 0.05 were selected for the analysis. As a result, 216 DE genes between early-mid, 157 between early-late and 219 between mid-late lactation stages were identified (Table S4).

### Early versus mid lactation

Comparison of the DE genes in early and mid stages of lactation revealed 76 upregulated and 140 downregulated genes. These DE genes could be classified into 130 functional categories with 87 GO terms for biological process, 22 terms for cellular components and 21 terms for molecular functions. Some of the significant (p < 0.05) GO terms for the upregulated genes were immune response, positive regulation of GTPase activity, lipopolysaccharide-mediated signaling pathway, transcription factor complex, chemokine activity and transcriptional activator activity. The downregulated genes were linked to regulation of cell proliferation, focal adhesion and calcium ion binding.

### Early versus late lactation

There were 125 upregulated and 32 downregulated genes across early and late lactation. The GO analysis revealed 47 terms for biological process, 7 terms for cellular components and 9 terms for molecular functions. Significant upregulated functions included immune response, cell adhesion, lipopolysaccharide-mediated signaling pathway, regulation of NFKB transcription factor activity, transcriptional activator activity, high-density lipoprotein particle assembly etc. The downregulated GO terms were synaptic vesicle fusion and SNARE complex.

### Mid versus late lactation

A total of 214 upregulated and 5 downregulated genes were observed on comparing mid and late lactation. The GO analysis categorized the genes into 88 biological processes, 40 cellular components and 15 molecular functions. Cell migration, positive regulation of transcription from RNA polymerase II promoter, branching involved in mammary gland duct morphogenesis, extracellular exosome, focal adhesion, calcium ion binding and growth factor activity were significantly enriched. The down regulated genes were associated with regulation of osteoblast differentiation as well as IL23-mediated signaling events.

### Interaction between DE genes

**Figure 5:**
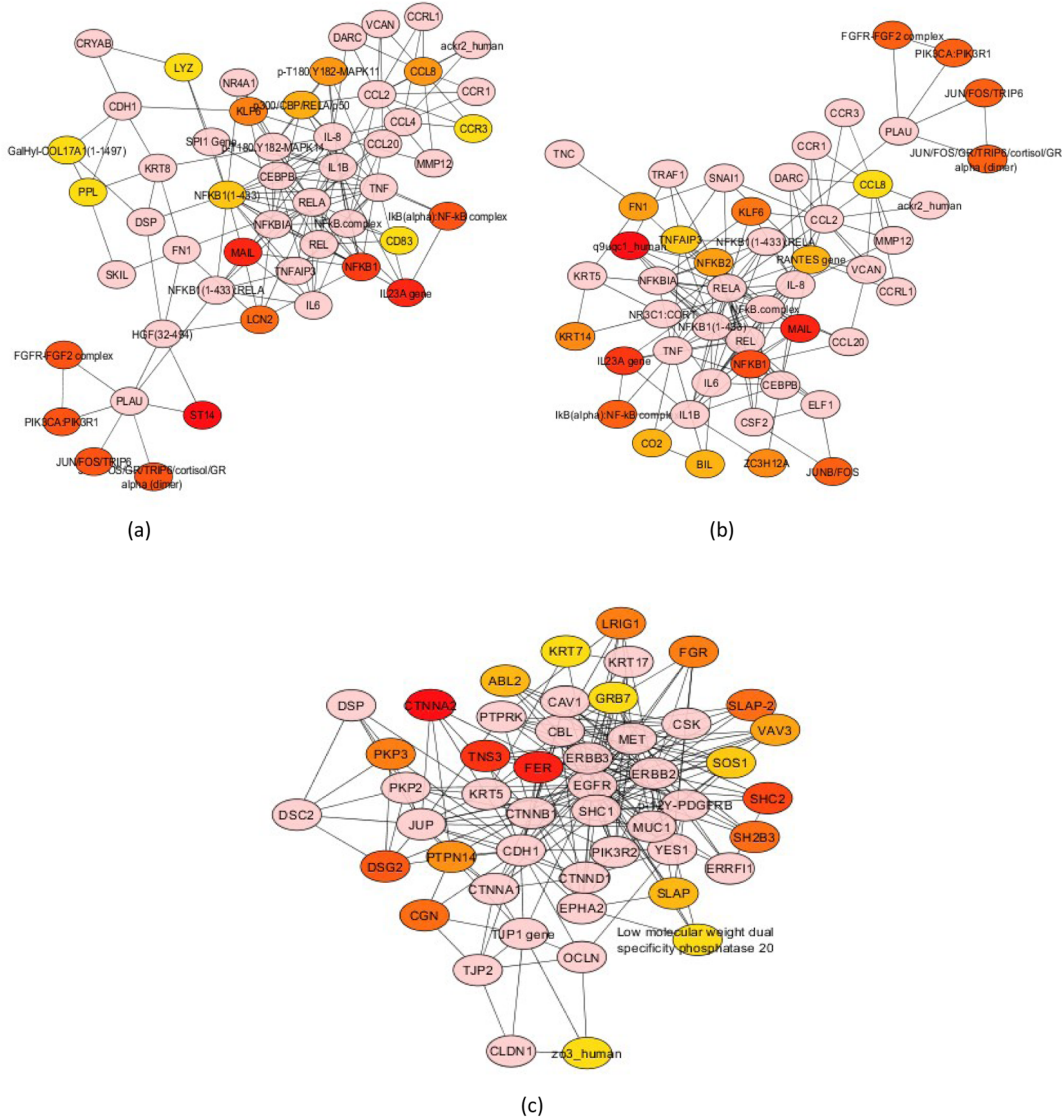
A subnetwork was used to enrich the interactions between the nodes, by selecting differentially expressed (DE) genes in the network, with ≥5.0 degree and a fold change of ≥2.0. The top 20 nodes ranked by Maximal Clique Centrality (MCC) scores were coloured orange to red. As the score decreases, the colour of the node changes from red to orange. (**a**) early-mid stage (**b**) early-late stage (**c**) mid-late stage.

The biochemical, protein-protein and gene regulatory interactions between the DE genes (FC ≥ ±2.0 and p_adj_ < 0.05), were analyzed by constructing a network from the DE genes between early-mid, early-late as well as mid-late stages. From the three primary networks, co-expressed genes with ≥5.0 degrees (having five or more interactions) were selected to construct sub-networks. The hub or highly connected genes between each stage were thus identified. *CCL8*, *CD83*, *LCN2*, *LYZ*, *MAIL*, *PPL* and *ST14* were identified from the root list as highly connected between early and mid lactation (Fig. 5a). The functionally important DE genes between the early and late stage were *CCL8*, *FN1*, *MAIL*, *NKFB2* and *Z3CH12A* (Fig. 5b). While comparison of the mid-late stage of lactation revealed *CGN*, *DSG2*, *GRB7* and *PTPN14* as hub genes (Fig. 5c). Among the highly connected DE genes, all except *LCN2*, *PPL* and *ST14* were upregulated.

### Validation of RNAseq data by qPCR

The differential expression of randomly selected genes like *CSN2*, *FABP3*, *LALBA*, *LPIN1*, *PAEP*, *RPS9* and *RPS23* was validated by qPCR. *ACTB* and *GAPDH* were used as the reference genes as they showed least variation in expression across the three stages in terms of RPKM (RNAseq data) and Ct values (qPCR). The validation was done by calculating the Pearson correlation between expression data of genes from RNA seq analysis and the expression data from qPCR. The expression pattern of these genes obtained by qPCR was in agreement to the RNAseq data although the magnitude was different (Table S5 and Supplementary Fig. 1).

## Discussion

Lactation is characterized with major physiological and metabolic changes in the individual. The early stage of lactation shows increased milk production, which peaks during the mid stage and starts declining towards the late stage^17^.To gain an insight into the expression of genes during lactation, we analyzed the transcriptome of milk from the early, mid and late lactation stages of Murrah buffalo. As established previously for goat and cattle^10,18^, the genes encoding the main milk protein, casein and whey (*CSN2*, *CSN1S1*, *CSN3*, *LALBA*, *PAEP* or *BLG*) were expressed abundantly (with RPKM > 3000) throughout lactation, with a slight decrease in the mid stage. The enhanced expression of these genes is relevant for the synthesis of milk proteins. The casein proteins account for up to 80% percent of the milk proteins while the whey proteins make up to 20%^19^. The milk proteins are synthesized in the mammary gland while some immunoglobulins cross over from the blood^20^. A significant decrease in protein and casein percent has been reported in the mid stage (4–5 months) as compared to early and late lactation stage in Murrah buffaloes^21^, as was also observed in our study. Many studies have shown that the highest concentration of proteins is present in the colostrum and early stage of lactation in different species^19,22^. *FTH1* and *SPP1* genes were almost equally expressed across the stages. *FTH1* codes for ferritin which has been reported in low quantities in bovine milk^23^. *SPP1* gene is also known to be involved in milk production and development of mammary gland^24^. Other genes that exhibited high expression (RPKM > 2000) in early and mid lactation were the ribosomal protein genes like *RPLP1*, *RPS11*, *RPSA* and *RPS8*. The increase in the translational machinery supports the synthesis of milk proteins during lactation. Interestingly, the *FABP3* gene was also among the top 20 highly expressed genes in the early stage. This gene has not been reported among the top expressed genes in goat and cattle^10,18^. As expected, most of the highly expressed genes in our study were responsible for biosynthesis of milk proteins.

Lactose is the most important carbohydrate in milk, which regulates the milk osmolarity^25^. Besides lactose, small amounts of glucose and galactose are also found in milk. α-lactalbumin encoded by *LALBA* regulates lactose production in the milk of most mammals^26^. It is an important gene of the lactose synthesis pathway. In our data *LALBA* was one of the top expressed genes across all stages with highest expression in the early stage (RPKM > 11000).

Milk fat is the major factor that determines the organoleptic quality as well as the commercial price of the milk. Buffalo milk has higher fat content than cow, goat or sheep^27^. Candidate genes reported to be associated with milk fat in other species^5^, were also observed in our study, with varied expression across the different lactation stages. Most of these genes had a higher expression in the late lactation stage (except *FABP4*) as compared to the mid stage. The late or advanced stage of lactation is marked by decrease in milk yield or volume which results in increase in the fat content^17^. The *FABPs* are involved in the uptake and transport of fatty acids. Over expression of *FABP3* has been linked to regulation of milk fat synthesis^28^ and increase in lipid droplet accumulation in cattle^29^. The sterol regulatory element-binding protein 1 (*SREBP1*) regulates triglyceride synthesis and also targets the *ACSS2* which is responsible for catalyzing the formation of short-chain fatty acids^30^ and *FASN* gene for catalyzing the formation of long-chain fatty acids in dairy cows^31^. While *PLIN2* and *XDH* are involved in the formation of lipid droplet^32^, their role in buffalo milk fat metabolism is not well elucidated.

Although most of the genes were expressed across all the three stages, a marked transition in GOs from early to late stage was observed. The early stage was mainly defined by protein metabolism which showed a decline in mid and late stages. The early lactation is characterized by increase in ribosomes and mitochondria due to enhanced energy requirement for protein synthesis^33^. The ribosomes associated with protein biosynthesis were mainly enriched in the early stage of lactation. Our results also reflect the molecular dynamics of lactation across the three stages.

Among the enriched pathways in our data, the GTP hydrolysis (GTPase) activity plays an important role in translation and translocation of proteins. GTPases are known to be involved in secretion of milk fat gobule^34^. The insulin pathway is reported to be involved in initiation of lactogenesis and regulation of milk secretion in humans^35^. These pathways are important for maintaining the high rate of protein biosynthesis in the mammary gland. These pathways underscore the importance of protein synthesis and modification during early and mid lactation. The phosphatidylinositol-3-kinase (*PI3K)* in conjunction with mTOR signalling pathway is known to regulate proliferation, metabolism and angiogenesis. Glypicans are cell surface molecules that regulate cell growth and morphogenesis^36^. Although some of these pathways have not been directly linked with lactation, they are known to regulate core cellular mechanisms inherent to lactation like proliferation, cell growth and metabolism. Most of the cellular machinery during lactation is involved in synthesis, modification, transport and secretion of milk proteins.

The physiological changes and stress during lactation affects the nutrient needs as well as the immune functions of the dairy animal^37^. Genes involved in immune response were present in all the stages but increased in the late stage. Various types of immune cells are involved during all stages of lactation from proliferation to involution. These include mast cells, macrophages, epithelial cells and endothelial cells, which not only provide immunity to the neonate but are also involved in development of the mammary gland^38^. During involution of mammary gland the immune response genes were over expressed in mice^39^. Our data also revealed enrichment of immune response in the late lactation stage of Murrah buffaloes. The percentage of genes associated with immune response increased from 10% in early stage to 23% in the late lactation stage. The immune cells help in removal of cell debris and milk in the late lactation stage^40^. Previous studies on bovine milk transcriptome have shown that the immune function genes were highly expressed in the late lactation stage^18^, which is in agreement with our results. The changes in gene expression across the different stages of lactation probably reflect the prominent changes occurring in the mammary gland during lactation.

We were able to identify highly connected DE genes between the three stages of lactation. The network analysis was utilized to understand the interactions between these DE genes. To simplify the complexity of interconnections, a subset of genes was considered in each case. Among the highly connected or hub genes in the early-mid stage comparison, *CD83*, *LCN2* and *MAIL* were regulated by *NFKB1*. *MAIL* is a member of the ankyrin-repeat family induced by lipopolysaccharide which promotes transcription of Lipocalin-2 (*LCN2*). *LCN2* is known to be involved in innate immune response as well as glucose tolerance and insulin sensitivity in mice and humans^41^. Between early and late stage, *NFKB2* and *MAIL* were connected to *NFKB1*. *NFKB* has been reported to play an important role during pregnancy and involution in mice^42^. The *NFKB* complex is expressed in various cells and plays an important role in the immune system as well as lactation and appears to be an important regulatory factor in buffalo lactation. Other hub genes *DSG2*, *FN1*, *GRB7*, *PPL*, *PTPN14*, *ST14* are associated with cell growth and adhesion, while *CCL8*, *CGN*, *LYZ*, *ZC3H12A* are linked with the immune system. The highly connected genes identified in our study are mainly implicated in immune response, cell growth and angiogenesis. Cell proliferation and growth of the mammary gland continues in the early and mid stage of lactation, while the immune cells may help in maintaining the health of the udder^43^. Further studies are required to verify the impact of the hub genes on the relevant pathways.

## Conclusion

This is the first study to describe the transcriptome profile of buffalo milk through the three lactation stages. The majority of the genes throughout lactation were involved in biological functions like protein metabolism, transport and immune response. There appeared to be a discernible shift from metabolism in early stage to metabolism and immune response in mid stage, and an increase in immune response in the late lactation. This shift is expected since the early lactation is characterized by high metabolic activity for the secretion of milk, which stabilizes in the mid stage and wanes towards the late stage. As a result, genes associated with the milk synthesis and secretions are activated during the early stage and their expression gradually declines in the late stage. Future research on the highly connected genes identified in our study may reveal their precise role during lactation.

## Materials and Methods

### Ethics statement

Ethical approval was not required for the study as no experiment was performed on the animals. Milk samples were collected from animals milked for commercial purpose. However, the project under which the study was conducted was duly approved by the Institutional Animal Ethics Committee, ICAR-National Bureau of Animal Genetic Resources, Karnal (F.No. NBAGR/IAEC/2017, dated 21.01.2017).

### Samples

Healthy multiparous buffaloes housed in the livestock farm of ICAR-NDRI, Karnal were selected. Four animals were selected for each group of early, mid and late lactation. The early stage of lactation included animals between 30 and 54 days postpartum, mid stage included animals between 117–136 days postpartum and late stage included animals between 250 and 273 days postpartum. All the animals were in the third parity with age between 6.5 and 7 years. Their milk yield ranged from 7–8 litres per day (Table S1). The milk samples were collected in summer (May–June), between 5:00–6:00 am, on the same day for each lactation stage. The samples were collected manually into sterile RNase-free tubes, taking care to avoid any RNase contamination. The samples were immediately placed on ice and analyzed within 4 hours.

### RNA isolation RNA sequencing

The non invasive methodology for RNA isolation from milk samples was used^44^. Approximately 50 ml milk was used for RNA extraction. Total RNA was purified using RNeasy kit (Qiagen). Four biological replicates from each stage of lactation, with RIN value ≥ 7.0 (Agilent Bioanalyzer) were used for library preparation by TruSeq RNA Library Prep Kit v2 (Illumina). Paired end sequencing of the 12 samples was performed on Illumina HiSeq-1000 Platform.

### Data analysis

Quality of the samples was assessed using FastQC (v 0.11.5). Trimming or filtering on raw reads was done using FastXToolKit according to the results of FastQC. CLS Genomics Workbench 6.5.1 (CLC Bio, Aarhus, Denmark) was used for data analysis. The trimmed reads were mapped to *Bubalus bubalis* (ftp://ftp.ncbi.nlm.nih.gov/genomes/Bubalus bubalis/), as well as *Bos taurus* genome (Btau 4.6). Expression levels of mapped reads were normalized as reads per kilobase million (RPKM) and reads with RPKM values < 0.01 were excluded from the study. PCA was carried out to verify the clustering of animals in the three groups using AltAnalyze v.2.1.0^45^. Venn diagram for distribution of transcripts was constructed using Venny^46^. Differential expression analysis was done using the CLC transcriptomics analysis tool, between different lactation stages in pairs (early-mid; early-late; mid-late). DE genes with log2 fold change ≥2.0, an adjusted p value (p_adj_) < 0.05 and an adjusted FDR < 0.05 were subjected to further analyses. Analysis of our data with both river buffalo and cattle reference assemblies gave similar results for gene expression as well as differential expression between the three stages. Therefore, all functional analyses of the genes were done using *Bos taurus* (Btau v4.6) as reference. The functional annotation and enrichment in pathways of the differentially expressed genes was carried out using DAVID^47,48^ and Consensus Pathway Data Base^49,50^. Cytoscape ver 3.6.0^51^ was used for network analysis along with Cytohubba app^52^.

### Validation by Real Time qPCR

The differential expression of some genes across the early, mid and late lactation stages was validated by qPCR. Primers sequences previously published for some random genes (*CSN*2, *FABP3*, *LALBA*, *LPIN1*, *PAEP*, *RPS9* and *RPS23;* Table S6) were used^28,53,54^. The total RNA extracted from milk was used for synthesis of cDNA. The cDNA was synthesized using 100 ng of purified RNA, 1 *μ*L 50 µM oligo (dT)20, 1 *μ*L 10 mM dNTP mix and nuclease free water to make the final volume 10 µl. The mixture was incubated at 65 °C for 5 min and kept on ice for 2 min. A total of 10 *μ*L of master mix composed of 2 *μ*L 10X RT Buffer, 2 *μ*L 0.1 M DTT, 4 *μ*L 25 mM MgCl_2_, 1 *μ*L of SuperScript III RT (200 U/µl), and 1 *μ*L of RNase OUT (40 U/µl) was added to cDNA mix. The reaction was performed in an Eppendorf Gradient cycler using the program: 50 °C for 50 min and 85 °C for 5 min. After chilling on ice for 5 min, RNase H (1 µl) was added to each tube and incubated for 20 min at 37 °C followed by PCR amplification. The qPCR reaction was performed in triplicate in a final volume of 10 µl containing 2 µl of cDNA, 8 µl of qPCR master mix (5 µl of SYBR Green Real-Time master mix, 0.3 µl of each primer, 2.4 µl of DNA/RNA-free water) on Roche Light cycler 480 system. PCR efficiency was estimated by standard curve calculation using four points of cDNA serial dilutions. The mean cycle thresholds (Ct) values of the genes were normalized to geometric mean of *ACTB* and *GAPDH* which were used as reference genes. The data was analyzed by the 2^−ΔΔCT^ method^55^.

## Supplementary information

Supplementary Figure 1

Dataset 1
