# Supplementary material for: Buffalo milk transcriptome: A comparative analysis of early, mid and late lactation
Source: Sci Rep. 2019 Apr 12;9:5993. doi: 10.1038/s41598-019-42513-2 (PMC6461664; doi:10.1038/s41598-019-42513-2)
Supplement: Supplementary file 1 — Supplementary Figure 1 [file 41598_2019_42513_MOESM1_ESM.pdf]

Supplementary Information

(Revised manuscript)

Buffalo milk transcriptome: A comparative analysis of early, mid and late lactation

Reena Arora*, Anju Sharma, Upasna Sharma, Yashila Girdhar, Mandeep Kaur, Prerna Kapoor Sonika Ahlawat and Ramesh Kumar Vijh

ICAR-National Bureau of Animal Genetic Resources, Karnal-132001, Haryana

***Corresponding author: rejagati@gmail.com**

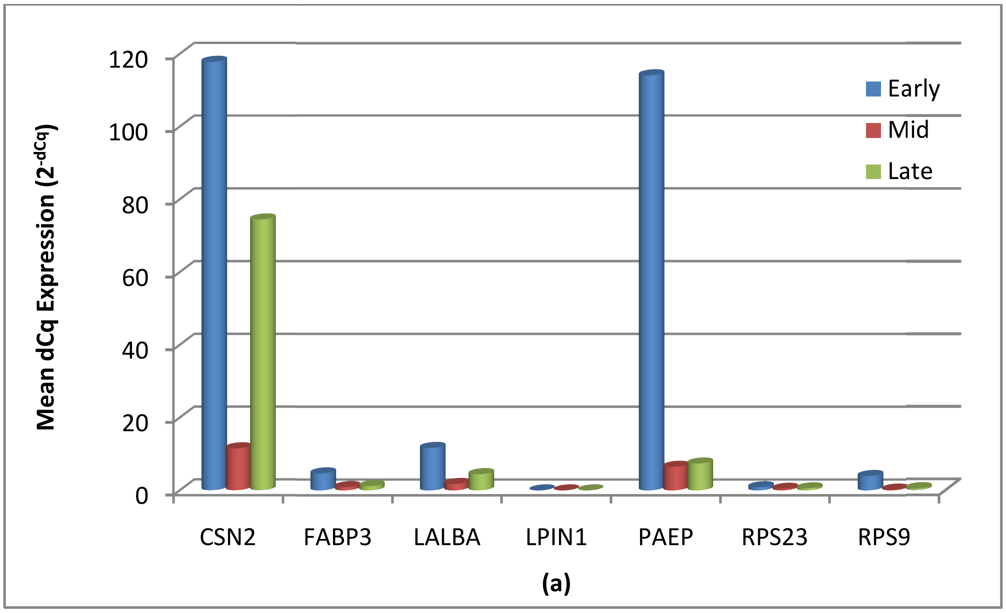

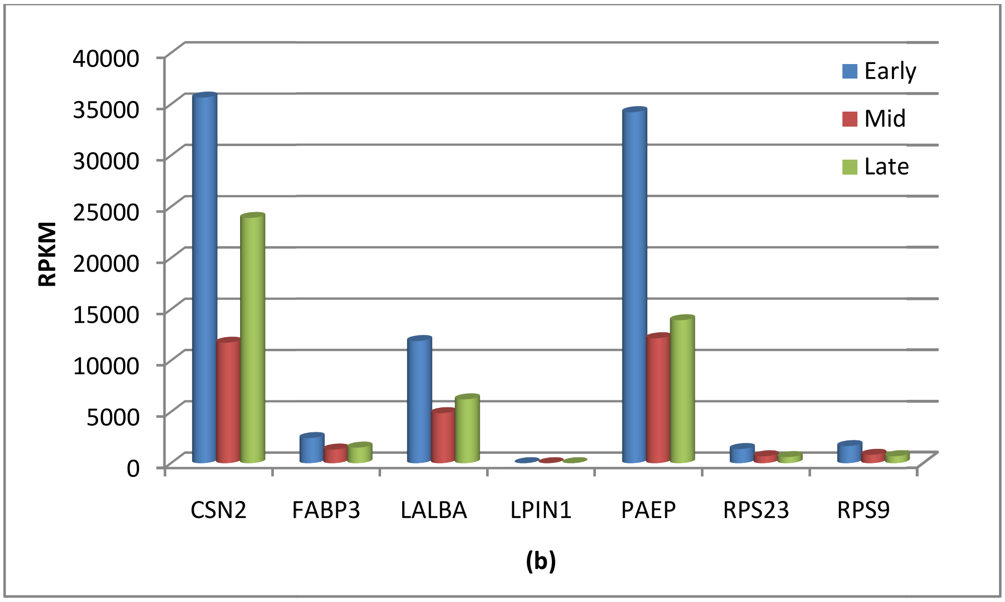

Supplementary Figure 1. Validation of DEGs by qPCR, (a) Mean dCq Expression values for selected genes (b) RPKM values for selected genes and reference genes
